# The management of vaginal prolapse and stress incontinence mesh complications in a quaternary mesh complications service in the United Kingdom (U.K): a 5-year observational study

**DOI:** 10.1186/s12905-025-03916-8

**Published:** 2025-08-02

**Authors:** Hawra Badri, Azita Rajai, Karen Ward, Richard Edmondson, Fiona Reid

**Affiliations:** 1 Warrell Unit, Saint Mary’s Hospital, Manchester University NHS Foundation Trust, Manchester, UK; 2 Manchester Academic Health Science Centre (MAHSC), Manchester, UK; 3 Division of Developmental Biology & Medicine, School of Medical Sciences, Faculty of Biology, Medicine and Health, University of Manchester, Manchester, UK; 4 Department of research and innovation, Manchester University NHS Foundation Trust, Manchester, UK; 5 Centre for Biostatistics, Faculty of Biology, Medicine and Health, University of Manchester, Manchester Academic Health Science centre, Manchester, UK; 6 Division of Cancer Sciences, School of Medical Sciences, Faculty of Biology, Medicine and Health, The University of Manchester, Manchester, UK; 7 Department of Gynaecological Surgery, Manchester University NHS Foundation Trust, Manchester, UK; 8 Division of Pharmacy and Optometry, School of Health Sciences, Faculty of Biology, Medicine and Health, University of Manchester, Manchester, UK

**Keywords:** Mesh complications, Pain, Stress urinary incontinence, Pelvic organ prolapse

## Abstract

**Objective:**

To describe the patient cohort accessing a quaternary-level pelvic mesh referral service, identify their requirements and ensure services can meet their needs. To determine areas of future research.

**Design:**

Retrospective and prospective observational study.

**Setting:**

Quaternary-level pelvic mesh complications service in the U.K.

**Population:**

Women accessing a pelvic mesh complications service over 5 years.

**Methods:**

All women attending the mesh complication service between 2018 and 2023 were included. Data was collected on referral rates, demographics, mesh complications experienced, management options selected, and post operative complications suffered. Mesh complications were compared against mesh devices and management options chosen.

**Results:**

785 women were managed in the mesh service over 5 years. Of 765 women with confirmed mesh devices, 92% (*n* = 707/765) were referred with a painful mesh complication and 54% (*n* = 416/765) reported pain alone. 58% requested surgical management (*n* = 403/692). Of 288 who received surgery, 52% (*n* = 150/288) requested complete mesh excision. The recurrent Stress Urinary Incontinence (SUI) and Pelvic Organ Prolapse (POP) rate was 66% (*n* = 141/215) and 23% (3/13) respectively. 18% (*n* = 51/288) experienced a surgical complication however only 0.7% (2/288) were considered serious.

**Conclusions:**

This study has identified patients reporting ‘pain alone’ as the commonest patient cohort attending a UK pelvic mesh centre between 2018 and-2023, suggesting that mesh associated pain exerts considerable burden on affected individuals and maybe more prevalent than once thought. This group has the greatest variation in treatment. Further research is required to understand the pathophysiology of mesh related pain to inform effective treatment options.

**Supplementary Information:**

The online version contains supplementary material available at 10.1186/s12905-025-03916-8.

## Introduction

Stress Urinary Incontinence (SUI) and Pelvic Organ Prolapse (POP) are common, affecting approximately one third and one half of women respectively [1].

Conservative management is often unsuccessful, leading 10% of women to request surgery [2]. Synthetic surgical mesh gained popularity due to its lightweight properties and durability making it ideal for reinforcing weakened tissue leading to its widescale uptake in hernia, POP, and SUI surgery.

Mesh procedures for SUI had advantages over existing procedures as they could be performed as day case operations and were associated with equivalent success rates. The Tension free vaginal Tape (TVT) became the gold-standard operation for SUI [3–5]and the commonest continence procedure performed in the UK.

Concerns regarding the safety of mesh products were raised by several agencies [6–8], and together with pressure from patient-led campaigners culminated in the suspension of vaginally inserted mesh in the UK and the commissioning of an independent parliamentary review of mesh complications. The recommendations of this review included the formation of specialist mesh centres and, since April 2021, nine centres in England have been commissioned to manage complications of SUI and POP devices [9].

The aim of this study was to describe the patient cohort seen in one of the largest mesh complication centres in the UK, describe the complications seen, method of assessment, and management pathways chosen by the patients. The findings of this study are intended to define the needs of this patient cohort, which will enable services to be designed to meet these needs and identify areas for future research.

## Method

All patients reviewed in the Manchester mesh complication service between 26/01/2018, and 19/04/2024 were included in the study. Data were collected from the electronic patient records. Data was collected retrospectively between 26/01/2018 before prospective data collection began on 19/10/2022 when a dedicated research fellow was employed to manage mandatory data collection for the service. All data were collected as part of routine clinical care hence Research Ethics Committee (REC) approval was not required.

Data collection included demographics, referral indications, management options chosen and surgical management details including extent of mesh excision, inpatient stay and complications. Data on mesh devices were extracted from referral letters or original operation notes. When these records were unavailable, patient’s account and the appearance of mesh devices on MRI and Trans Labial Ultrasound (TLUSS), were used to determine the type of mesh inserted.

Comorbidities were classified into three groups (see supplementary Table 1). Conditions relevant to more than one category are listed multiple times. These conditions were selected due to their potential relationship with mesh complications: pain and mood disorders relating to experience of pain and concerns amongst mesh affected patients regarding the relationship between autoimmune conditions and mesh within their body.

Mesh complications were categorized according to the International Urogynaecology Association (IUGA) and International Continence Society (ICS) classification [10]. Vaginal exposure or extrusion describes mesh becoming revealed in the vagina. Perforation refers to mesh passage out of a structure into another including bladder, urethra or bowels.

Mesh infection was determined by examination findings; local tenderness, erythema, presence of purulent discharge, an abscess or sinus tract or radiological findings of a collection or thickening around a device. Visceral perforation was diagnosed using cystourethroscopy in women with SUI mesh and MRI in women with abdominal mesh. Patients with mesh infections, perforations or vaginal exposures exceeding 1 cm were counselled to undergo mesh excision as recommend by national guidance [1]. Voiding and urodynamic studies were performed to investigate bladder emptying issues and urinary incontinence.

Patients reporting pain without mesh exposure, perforation or infection were defined as having “pain alone”.

Management recorded were those selected by patients following detailed counselling. Non-surgical management included pharmacological treatments, (vaginal oestrogen, oral and transdermal analgesia, steroid or local anaesthetic injections), psychological (psychotherapy, psychosexual counselling, and group pain therapy) and physiotherapy.

Surgical management included mesh trimming, division, and partial or complete excision. Excision of mesh remnants previously incompletely removed were classified as complete mesh removals. Those wishing additional time to consider their options, were recorded as ‘undecided’. Patients who declined treatment and follow up following assessment were recorded as ‘counselled and discharged’.

Patients were grouped by mesh device: SUI devices referring to retropubic and trans-obturator tapes (TOT), vaginal POP mesh devices and abdominal mesh. Patients with more than one mesh device were categorised as having ‘multiple’ devices. Patients were also grouped based on the principal diagnosis (Table 1).

## Results

### Design of the service

**Fig. 1:**
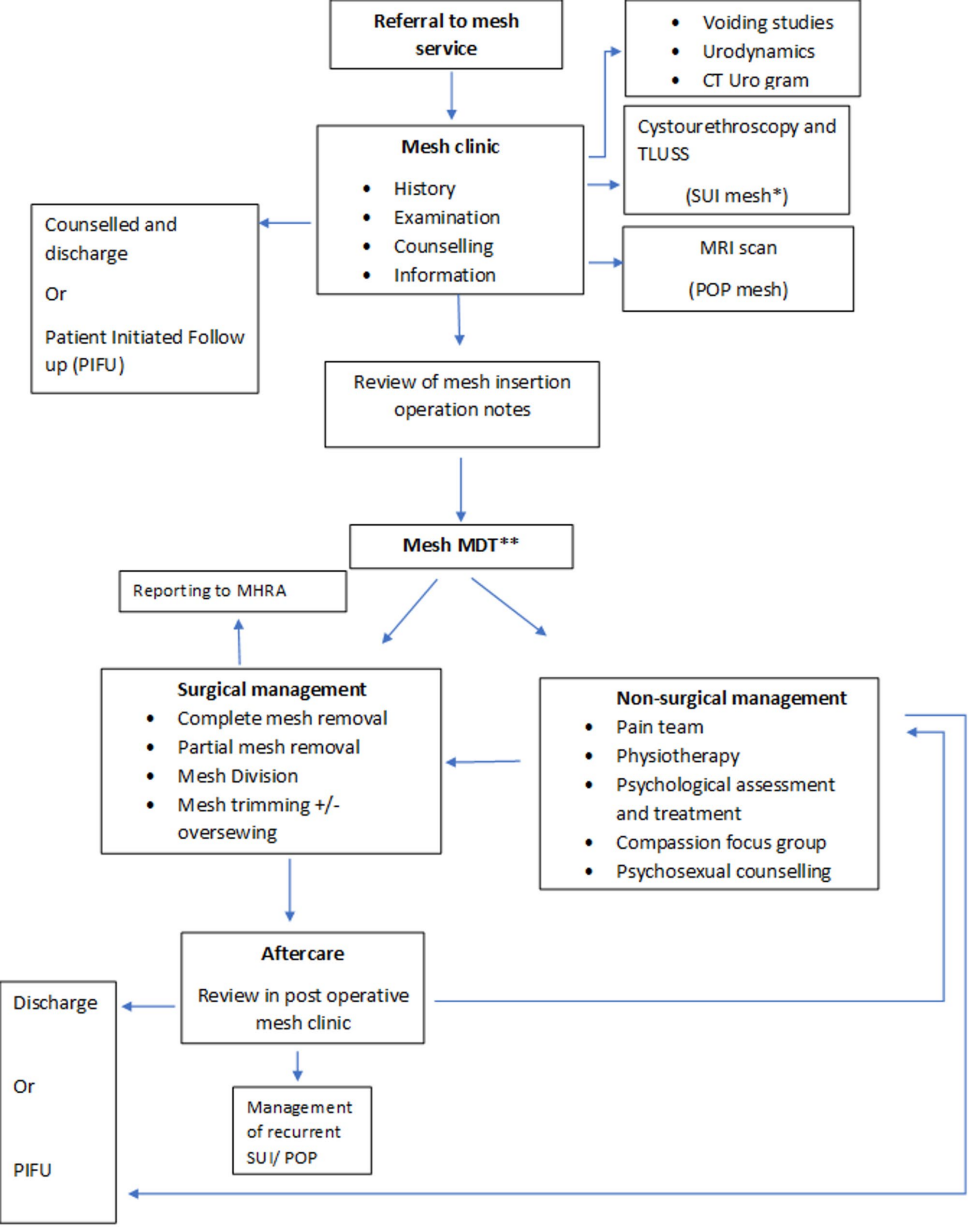
Management pathway for patients presenting to mesh complications service. *Patients with POP devices may also be investigated with cystourethroscopy when clinically indicated. **Mesh MDT comprises a minimum of two urogynaecologists, a urologist, a colorectal surgeon, a plastic surgeon, a radiologist, a psychologist, a psychosexual therapist, pain specialist, urogynaecology clinical nurse specialists, and a pelvic health physiotherapist

The Manchester mesh complication service was established in 2016. Recognising the complexity of these complications, dedicated mesh clinics were established from 26/01/2018. The service is amongst the largest in the UK. The members of the Multidisciplinary Team (MDT) are listed in Fig. 1. Patients experiencing complications of SUI devices are reviewed in a one- stop service allowing provision of investigations and counselling in a single visit. Detailed assessments include history, standardised questionnaires, examination, cystourethroscopy and imaging including MRI or Trans labial ultrasound (TLUSS). Holistic and individualised care is provided. Management plans are based on the principles of shared decision-making. Aftercare following treatment is an essential component of the service. The management pathway is detailed in Fig. 1.

### Referral patterns

785 patients were managed at in the service over 5 years. 20 patients had unconfirmed mesh devices at the time of analysis and were excluded from the study.

SUI mesh devices accounted for 71% (*n* = 545/765) of all devices. Complications of vaginal POP mesh devices were the least common contributing to 7% (*n* = 50/765) of referrals.

**Fig. 2:**
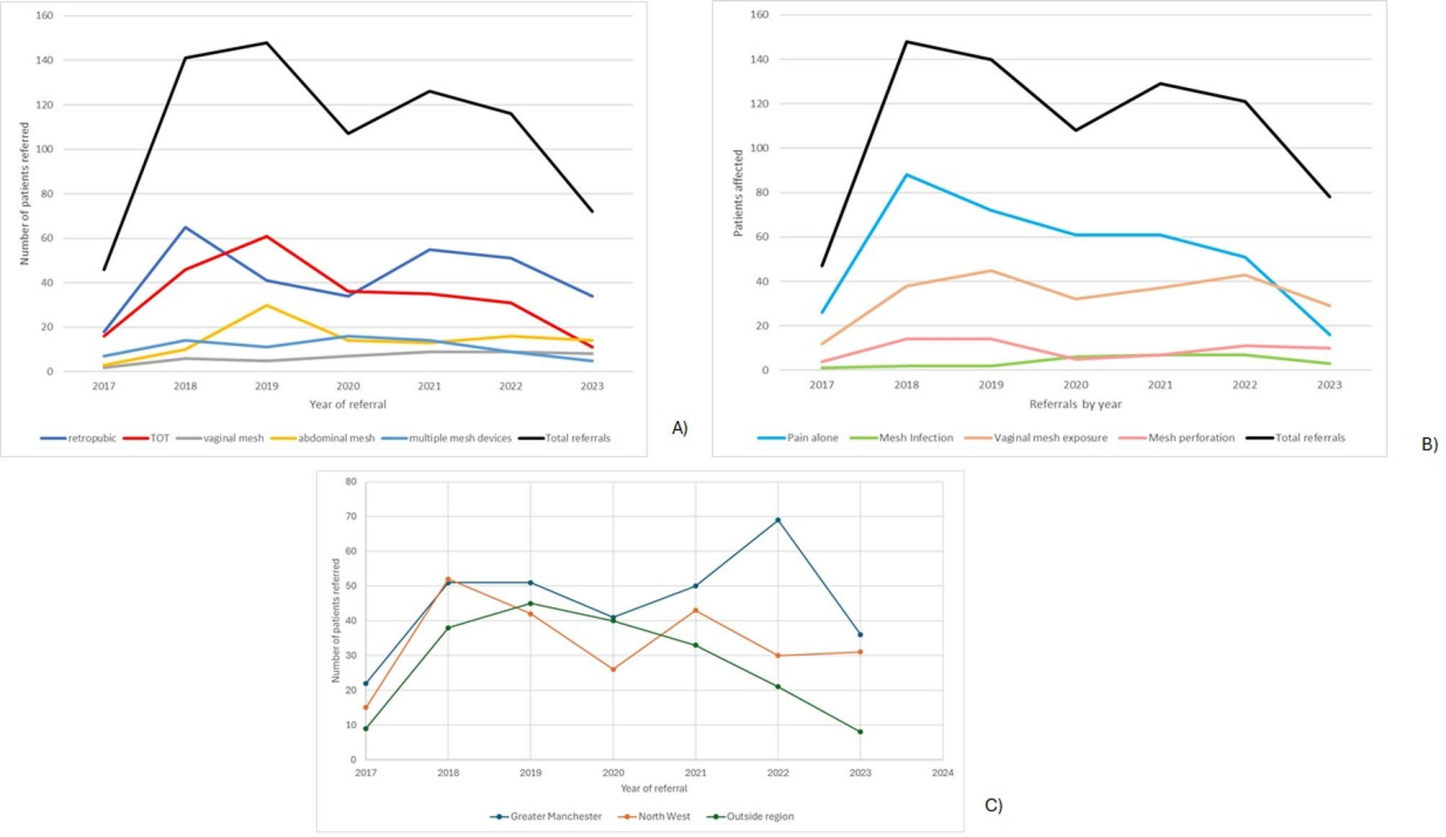
(**A**) Referrals to the mesh complications service by mesh devices per year. (**B**) Referrals to the mesh complications service by mesh complication per year. (**C**) Referrals to Manchester mesh complications service per year from within Greater Manchester, the Northwest and outside of this region

Referrals to the service per year by mesh device are illustrated in Fig. 2 (A). Referrals for vaginal POP mesh complications remained consistent across the 5 years. Total referrals had a bimodal appearance, with a peak in 2019 and a further peak in 2021.

Referrals to the service from outside the region have declined steadily over 5 years, but referrals from the Northwest region have remained consistent after 2020. (See Fig. 2 (C)).

### Patient characteristics

Demographic details for each device group are detailed in Table 1. Patients’ median BMI, across all mesh devices were close to the obese range.

The rates of co-existing chronic pain and autoimmune conditions of unknown aetiology were similar across all mesh devices and diagnoses.

**Table 1:** Demographic details for patients seen in mesh complications service by mesh device and complications

Total Referrals	Retropubic (*n* = 304) (39%)	TOT (*n* = 241) (31%)	Vaginal POP (*n* = 50) (6%)	Abdominal (*n* = 92) (12%)	Multiple mesh (*n* = 78) (10%)	Pain alone (*n* = 416) (54%)	Vaginal exposure (*n* = 245) (32%)	Visceral perforation (*n* = 69) (9%)	Mesh infection (*n* = 35) (5%)
*N* = 765									
Age Median[range](IQR)	60[38–94] (55-67.5)	58 [31–92] (52–64)	71 [40–89] (61–77)	63 [36–91] (52.3–72.8)	61 [37–89] (54.25-70)	59 [31–94] (52–66)	62 [41–90] (56–70)	62 [41–90] (56–70)	63 [42–87] (55.5–71.8)
BMI Median [range] (IQR)	38 [19–54] (26-33.5)	29 [19–47] (26–34)	30 [20–40] (26–33)	28 [15–40] (24–31)	29.5 [21–46] (26.8–36)	30 [15–47] (26–34)	29 [19–44] (25.5–32)	29 [19–44] (25.5–32)	27 [ 21–38] (23–31)
Smoking	37 (12%)	23 (10%)	7 (14%)	6 (7%)	6 (8%)	34 (8%)	34 (14%)	5 (7%)	7 (20%)
Pain conditions	120 (39%)	126 (52%)	24 (48%)	29 (32%)	35 (45%)	202 (49%)	92 (38%)	35 (52%)	13 (37%)
Mood conditions	46 (15%)	38 (16%)	7 (14%)	9 (10%)	16 (21%)	55 (13%)	45 (18%)	9 (13%)	6 (17%)
Autoimmune conditions	41 (13%)	29 (12%)	8 (16%)	16 (17%)	14 (18%)	55 (13%)	33 (13%)	9 (13%)	2 (6%)
Diabetes	16 (5%)	21 (9%)	5 (11%)	5 (10%)	6 (8%)	37 (9%)	11 (4%)	5 (7%)	4 (11%)
Index of deprivation Median (IQR)	5 (2–8)	5 (2.3-8)	5 (4–7)	6 (3–8)	5 (3–7)	5 (3–5)	5 (2–8)	4 (2-6.3)	5 (2–7)

### Indications for referral

Pain was the commonest complaint affecting 92% of patients (*n* = 707/765). Pain alone accounted for over half (54% *n* = 416/765) of referrals.

Eight- percent of patients had painless mesh complications (*n* = 58/765). 13% (*n* = 33/245) experienced a pain-less vaginal mesh exposures, 19% (*n* = 13/69) a pain-less mesh perforations and 34% (*n* = 12/35) were revealed to have a mesh infection without associated pain.

**Fig. 3:**
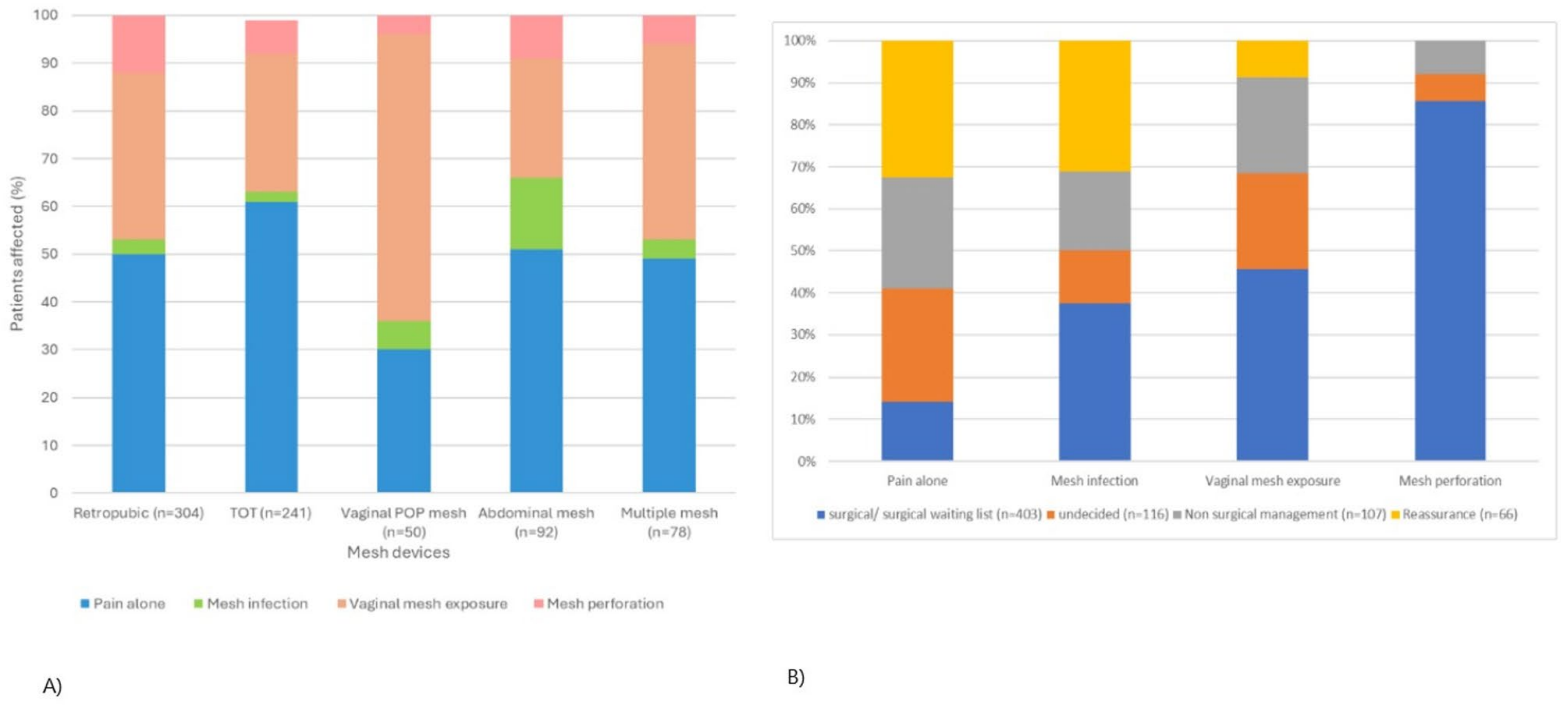
(**A**) The incidence of principal mesh complications by mesh device. (**B**) Management choices selected by patients following counselling and investigation by principal mesh complication experienced

The incidence of mesh complications by mesh device are represented in Fig. 3 (A).

Unlike other devices the commonest complication of vaginal POP was vaginal exposure of mesh, accounting for 60% (*n* = 30/50) of complications experienced with this mesh device.

Nine- percent (*n* = 69/765) experienced visceral mesh perforation. Urethral perforations were the commonest accounting for 59% (*n* = 41/69), followed by bladder perforations 14% (*n* = 10/69). Bowel perforations occurred in 6% of cases (4/69).

Urethral mesh perforation occurred at similar rates in retropubic and TOT devices (8.2% and 6.6%).

### Management patterns

Of the 765 patients, 7 died of unrelated conditions before confirming treatment desired, 5 transferred their care to a mesh service closer to home and 61 patients were still undergoing investigations.

The management option chosen by the remaining 692 patients are highlighted in Fig. 3 (B).

Most patients opted to receive treatment (74% *n* = 510/692), but a proportion were assessed, reassured and did not require or opt for treatment. These patients were offered long term surveillance or “Patient Initiated Follow Up” (PIFU).

### Surgical management of mesh complications

Surgical management was requested by 58% (403/692) of patients. Of the 403 patients who chose a surgical intervention, to date 288 had received it. Surgical management categorised by mesh device is detailed in supplementary Table 2.

In total, 53% (*n* = 152/288) of patients opted to have complete mesh excision. Forty patients (26% *n* = 40/152) within this group had removal of a mesh remanent incompletely removed at a previous surgery.

Most abdominal mesh removals were performed laparoscopically (81% *n* = 26/32).

Concomitant continence surgery was performed following detailed patient counselling and MDT approval in patients with bothersome urodynamic SUI who requested it. A concomitant continence procedure was performed in 30 of the 215 (14%) patients having SUI mesh removal (73% (*n* = 22/30) underwent autologous fascial sling, 17% (*n* = 5/30) laparoscopic Colposuspension and 10% (*n* = 3/30) urethral bulking. Forty patients (19% *n* = 40/215) underwent continence surgery at a later date; (68% (*n* = 27/40) autologous fascial sling, 13% (*n* = 5/40) laparoscopic Colposuspension and 20% (*n* = 8/40) urethral bulking).

Patients undergoing surgical excision of TOT devices had a higher rate of recurrent SUI (74% *n* = 78/106) than those undergoing removal of a retropubic device (58% 63/109). However, patients with TOT devices had a higher incidence of complete mesh excision than those with retropubic devices (71% *n* = 75/106 versus 40% *n* = 44/109).

Recurrence of POP occurred in 23% (*n* = 3/13) of patients following vaginal POP mesh removal and in 38% (*n* = 12/32) following removal of abdominal prolapse mesh.

Complications varied depending on the mesh device (See supplementary Table 2). Serious post operative complications (Clavien Dindo > 2) were uncommon (*n* = 2 /288 0.7%). However, 2 patients required a stoma as part of their abdominal mesh removal.

## Discussion

### Main findings

Our study identified pain as the commonest mesh complication referred to a quaternary mesh service over 5 years with pain alone contributing to over half of referrals. Surgical management was the commonest treatment requested, with complete mesh excision requested by over half of patients. Serious complications of mesh excision were low however risks of recurrent SUI and POP were high. These findings highlight that pain associated with mesh devices is prevalent and burdensome. Considering this, mesh services managing patients reporting mesh associated pain should ensure patients receive MDT input, be offered a complete range of non-surgical and surgical management options and receive accurate, informed and realistic counselling on predicted success of surgery to alleviate pain together with the risk of complications and recurrent SUI and POP rate. Further research is required to understand the aetiology of mesh related pain, to examine the efficacy of surgical and nonsurgical management in alleviating pain and to explore impact of pain on quality of life together with patient motivations and expectations of surgery.

Referrals to the Manchester mesh complications service peaked in 2019. Media attention surrounding the national mesh pause in July 2018 and heightened awareness of mesh complications is a likely explanation for this. The impact of the COVID-19 pandemic on non-emergency healthcare provision likely accounted for the reduction in referrals in 2020. Over the 5 years referrals to the service have declined, mainly due to a reduction in those referred with pain alone. Referrals of patients with mesh exposure and perforation have remained constant. There are several potential explanations for this. The formal commissioning of mesh centres by NHS England in 2021 is likely to have resulted in patients being referred to centres closer to home. Figure 2 C shows a consistent decline in referrals from outside of the Northwest region however analysis of national mesh centre referral patterns is needed to confirm this. Long waiting times for assessment and surgery may have deterred some patients from accessing care within the NHS and led them to self-fund private care in the UK and abroad [11]. Recognition of mesh complications remains low within healthcare services and there may be an under referral of eligible patients in view of reduced media focus. One service in Australia [12], saw a large increase in referrals when patients were contacted directly by letter.

The incidence of chronic pain secondary to mesh devices is variable. One systematic review reports an incidence of 1% in SUI devices and 7% in POP devices [13], elsewhere in the literature, rates as high as 30% [14] are reported following mesh-based pelvic floor surgeries. Our service identified ‘pain alone’ as the commonest complication, with over half of patients (54% *n* = 416/765) referred with this as a principal diagnosis. This suggests pain related to mesh devices maybe more prevalent that previously known. Pain is the leading cause of litigation for mesh complications in the USA [15] and reflects the impact it has on patients’ quality of life. It is possible that mesh pain is more widespread than currently recognised due to difficulties in defining and assessing pain, compared to conditions such as exposure or perforation. However, pain following all surgical procedures is common, with a reported 50% risk of surgery provoking de novo pain [16]; differentiating between surgical induced pain from mesh associated pain is challenging.

Mikilos et al. [17] reported a cohort of 445 patients undergoing mesh revision surgery, in three centres in the USA, from 2011 to 2013. They also found pain to be the most common complaint. However, the study reported more patients who had complications following POP mesh than continence meshes compared to our study (44% versus 19%). The study did not provide any context of those who did not require surgical intervention.

In our service surgical management was the leading treatment selected by 58% (*n* = 403/692) of patients. Many patients with suspected mesh infection, visceral mesh perforation and vaginal mesh exposure exceeding 1 cm were actively counselled to have surgery based on guidance [1] influencing this finding.

Incomplete mesh removal with remnants remaining within the groin and abdominal cavity can be more difficult to identify than when the mesh is complete. Additionally, repeat attempts at abdominal mesh removal carry risks of vascular and visceral injury with increasing risk encountered with abdominal adhesions. Considering this, surgeons may counsel patients with abdominal or TOT devices towards complete excision. This is reflected in the higher levels of complete removal of TOT devices compared to retropubic (71% versus 40%).

Another mesh complication service in Australia [12] reported only 36% of their patients chose a surgical management option. This may reflect the different way in which patients access the two services. In Queensland all patients who had had mesh were contacted by post and informed of the mesh complications service. In the UK patients must present via a GP. This could reduce access to care for patients in the UK. Only 9% (*n* = 66/765) of patients in the Manchester Service were assessed and discharged with no further intervention suggesting a more severe group of symptoms seen in our service.

Media reports suggesting mesh may cause a foreign body effect and autoimmune conditions [18] could be responsible for patients requesting complete mesh excision. However, there were similar rates of autoimmune disorders in this patient cohort (14% *n* = 108/765) as are reported in the UK population (13%) [19].

In our service patients presenting with pain alone requested a range of non-surgical treatments following assessment and counselling. This included counselling and treatment by a pain specialist, physiotherapist, psychologist and psychosexual counsellor. Realistic counselling on the uncertainty of surgery resolving pain is likely to be responsible for this pattern together with concerns about recurrent SUI and POP following mesh excision. These findings suggest that despite the apparent disposition for this patient cohort to request surgery, there is willingness to consider alternative management options when fully informed about risks and alternative therapies available.

The study found a higher proportion of patients with chronic pain comorbidities, not mesh related, than the 30% expected UK prevalence rates [20]. However, we were unable to determine whether these conditions pre-dated mesh insertion due to lack of documentation and difficulties in patients recalling when symptoms began.

### Future research

Aetiology of pain following mesh insertion is poorly understood and further research is required to understand the pathophysiology of mesh related pain. Exploration of pain outcomes following complete and partial mesh removal is required to guide decision making in this area. Factors influencing patient decision making are unknown. Some patients state a psychological benefit from the knowledge that all mesh has been removed [21]. Qualitative research to explore factors influencing patient decision making regarding treatment choices would further support understanding on how to ensure our services meet our patient’s needs.

### Strength and limitations

The strength of this study is the large, 765 patients, cohort managed by a single service over 5 years. This is the first comprehensive report from a commissioned mesh centre in the U.K. The study presents detailed and consecutive data from a full range of SUI, POP and multiple mesh devices managed in the service. We acknowledge the limitations of the study including inclusion of retrospectively collected data. Data on proportion of patients whose mesh devices were confirmed by imaging and examination findings was also lacking. Clinical outcome data from the service is presented elsewhere [19].

## Conclusion

This study has identified patients reporting ‘pain alone’ as the commonest patient cohort attending a UK pelvic mesh centre between 2018 and 2023, suggesting that mesh associated pain exerts considerable burden on affected individuals and maybe more prevalent than once thought. This group has the greatest variation in treatment. Further research is required to understand the pathophysiology of mesh related pain to inform effective treatment options.

## Electronic supplementary material

Below is the link to the electronic supplementary material.

Supplementary Material 1

## Data Availability

The data that support the findings of this study are not openly available due to reasons of sensitivity and are available from the corresponding author upon reasonable request.

## Abbreviations

SUI: Stress urinary incontinence
POP: Pelvic organ prolapse
TVT: Tension free vaginal tape
TOT: Trans obturator tape
TLUSS: Trans labial ultrasound
MDT: Multi-disciplinary team
