# Supplementary material for: The management of vaginal prolapse and stress incontinence mesh complications in a quaternary mesh complications service in the United Kingdom (U.K): a 5-year observational study
Source: BMC Womens Health. 2025 Aug 2;25:381. doi: 10.1186/s12905-025-03916-8 (PMC12317572; doi:10.1186/s12905-025-03916-8)
Supplement: Supplementary file 1 — Supplementary Material 1 [file 12905_2025_3916_MOESM1_ESM.docx]

Supplementary

Table 1: Classification of co morbid conditions.

| Pain related conditions | Mood related conditions | Autoimmune conditions |
| --- | --- | --- |
| Fibromyalgia  Polymyalgia  Complex Regional Pain  Irritable Bowel Syndrome (IBS)  Diverticular disease  Inflammatory Bowel Disease  Gallstones  Renal Stones  Chronic back pain  Lichen Sclerosis  Endometriosis  Adenomyosis  Rheumatoid Arthritis  Osteoarthritis  Migraines  Pelvic Congestion Syndrome  Burning mouth syndrome | Anxiety and Depression  Schizophrenia  Bipolar disorder  Post Traumatic Stress Disorder (PTSD)  Personality disorder | Lichen Sclerosis  Rheumatoid arthritis  Myasthenia Gravis  Hypothyroidism  Chronic fatigue  Burning mouth syndrome  Psoriasis  Sjogren’s syndrome  Sarcoidosis  Barrett’s Oesophagus  Vulval Intraepithelial Neoplasia (VIN)  Coeliac’s disease  Lupus  Hidradenitis Suppurativa |

Table 2: Surgical management of mesh devices including length of inpatient hospital stay, and complications experienced following surgical management of mesh devices.

| Surgical management of mesh devices | Retropubic (n=109) (38%) | TOT (n=106) (37%) | vaginal mesh (n=13) (5%) | Abdominal mesh (n=32) (12%) | Multiple mesh devices (n=28) (10%) |
| --- | --- | --- | --- | --- | --- |
| (n= 288) |  |  |  |  |  |
| Complete removal | 44 (40%) | 75 (71%) | 2 (15%) | 19 (59%) | 10 (36%) |
| Partial removal | 65 (60%) | 28 (26%) | 11 (85%) | 13 (41%) | 17 (61%) |
| Mesh division | 0 | 3 (3%) | 0 | 0 | 1 (3%) |
| Length of inpatient stay and complications |  |  |  |  |  |
| Median inpatients stay (days) | 2.2 [0 to 23] | 2.9 [1 to 9] | 3.3 [1 to 14] | 3.7 [1 to 15] | 4.4 [1 to 20] |
| Wound infection/ haematoma | 6 (6%) | 13 (12%) | 0 (0%) | 1 (3%) | 5 (18%) |
| Urinary tract infection (UTI) | 0 (0%) | 1 (1%) | 0 (0%) | 1 (3%) | 0 (0%) |
| Prolonged catheterisation | 5 (5%) | 1 (1%) | 1 (8%) | 0 (0%) | 1 (4%) |
| Intra-abdominal collection | 0 (0%) | 0 (0%) | 0 (0%) | 2 (15%) | 1 (4%) |
| Bladder/ bowel injury | 1 (1%) | 2 (2%) | 0 (0%) | 2 (6%) | 0 (0%) |
| Stoma formation | 0 (0%) | 0 (0%) | 0 (0%) | 2 (6%) | 1 (4%) |
| chest infection | 1 (1%) | 1 (1%) | 0 (0%) | 0 (0%) | 1 (4%) |
| Incisional hernia | 0 (0%) | 0 (0%) | 0 (0%) | 1 (3%) | 1 (4%) |
| Total complication rates (%) | 13 (12%) | 18 (17%) | 1 (8%) | 9 (28%) | 10 (36%) |
